# A Retrospective Study of Risk Factors for Symptomatic Anastomotic Leakage after Laparoscopic Anterior Resection of the Rectal Cancer without a Diverting Stoma

**DOI:** 10.1155/2020/4863542

**Published:** 2020-04-13

**Authors:** Zhi-Jie Wang, Qian Liu

**Affiliations:** Department of Colorectal Surgery, National Cancer Center/National Clinical Research Center for Cancer/Cancer Hospital, Chinese Academy of Medical Sciences and Peking Union Medical College, Beijing 100021, China

## Abstract

**Background:**

Anastomotic leakage (AL) is a common and devastating postoperative issue for patients who have undergone anterior resection of rectal carcinoma and can lead to increased short-term morbidity and mortality. Moreover, it might be associated with a worse oncological prognosis of tumors. This study is aimed at exploring the risk factors for symptomatic AL after laparoscopic anterior resection (LAR) for rectal tumors without a preventive diverting stoma.

**Materials and Methods:**

This case control study retrospectively reviewed the data of 496 consecutive patients who underwent LAR of the rectum without a preventive diverting stoma at the Cancer Hospital, Chinese Academy of Medical Sciences between September 2016 and September 2017. All patients were divided into an AL group and a control group based on the occurrence of postoperative symptomatic AL. Factors regarding patient-related variables, operation-related variables, and tumor-related variables were collected and assessed between the two groups through univariate and multivariate logistic regression analyses to identify independent risk factors for AL.

**Results:**

In total, 18 (3.6%) patients developed postoperative symptomatic AL. Univariate analysis showed that a synchronous primary malignancy of the left hemicolon (*P* = 0.047), intraoperative chemotherapy (*P* = 0.003), and level of anastomosis (*P* = 0.033) were significantly related with AL. Multivariate analysis was subsequently performed to adjust for confounding biases and confirmed that a synchronous primary malignancy of the left hemicolon (odds ratio (OR), 12.225; 95% confidence interval (CI), 1.764-84.702; *P* = 0.011), intraoperative chemotherapy (OR, 3.931; 95% CI, 1.334-11.583; *P* = 0.013), and level of anastomosis (OR, 3.224; 95% CI, 1.124-9.249; *P* = 0.030) were independent risk factors for symptomatic AL for patients who received LAR for rectal neoplasms without a preventive diverting stoma.

**Conclusions:**

Synchronous primary malignancy of the left hemicolon, intraoperative chemotherapy, and a low anastomotic level can increase the risks of postoperative symptomatic AL after LAR of the rectum without a protective diverting stoma.

## 1. Introduction

**Figure 1:**
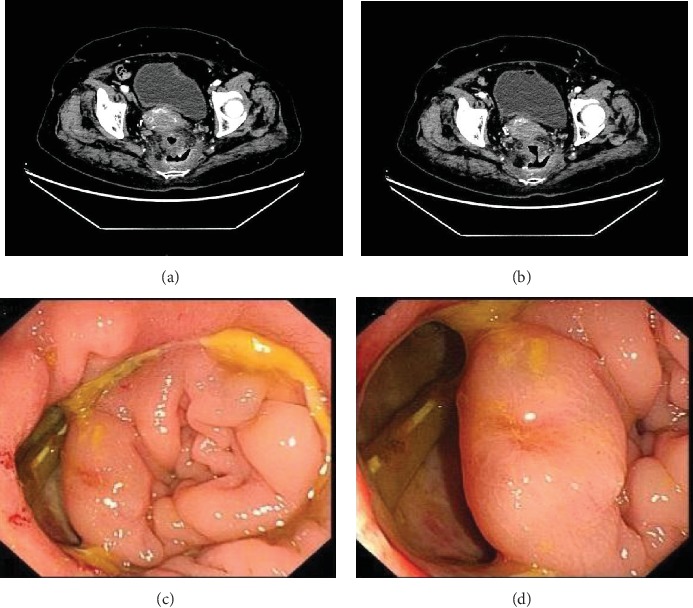
Images of anastomotic leakage. (a) Pelvic contrast-enhanced computed tomography picture showing pneumatosis and hydrops around the anastomosis; (b) pelvic contrast-enhanced computed tomography picture showing the communication between the lumen of the bowel and pelvic cavity; (c) endoscopic picture showing the defect of anastomosis; (d) endoscopic picture showing that the pelvic drainage tube can be seen from the anastomotic defect.

Anastomotic leakage (AL) is defined as a defect of the intestinal wall integrity at the anastomotic site, which leads to communication between the lumen of the bowel and the pelvic cavity (Figure 1). It is a major and serious surgical complication after anterior resection of rectal carcinoma, with the reported incidence varying considerably from 2.2% to 18.6% [1–3]. AL is associated with a prolonged hospital stay, increased medical costs, and a high occurrence of morbidity and mortality in a short time. Moreover, patients who developed AL can have poor long-term anorectal function resulting from pelvic fibrosis, which includes decreased maximum tolerated volume, increased fecal frequency, urgency, and incontinence [4]. More seriously, AL can also promote pelvic recurrence and decrease the overall survival, as it contributes to the spread of intraluminal residual tumor cells and local inflammatory related immunosuppression and delays postoperative adjuvant therapy [5, 6].

Patients with symptomatic AL usually present with symptoms and signs of fever, abdominal pain, peritonitis, and fecal discharge from the pelvic drainage [7]. Pelvic CT scans can show pneumatosis and hydrops around the anastomosis in the pelvic cavity. Based on the proposal by the International Study Group of Rectal Cancer in 2010, rectal AL can be classified as grades A, B, and C. Patients with grade A AL need no medical interventions, patients with grade B AL need only conservative treatment, and patients who develop grade C AL require a secondary operation. Symptomatic AL includes both grade B and C AL [8].

Many previous clinical studies have explored the risk factors and mechanisms of AL; however, most of these studies enrolled all patients who underwent laparotomy or laparoscopic surgery and all patients who received a protective stoma, which might lead to considerably different conclusions [1, 9, 10]. Laparoscopic surgery comprises a growing percentage of rectal surgeries and has been the main operation method in many countries and areas. Rectal surgery is usually difficult due to an insufficient operative view and limited working space in the pelvic cavity. The laparoscopic technique can provide a better operative field to facilitate surgery, but it also increases the difficulty of rectal transection because it is more difficult to provide adequate traction and effective cutting angles for the endolinear surgical stapler, unlike with open surgery. Therefore, the risk factors of AL may differ between laparotomy and laparoscopic surgery. Moreover, a protective diverting stoma can significantly prevent the occurrence of symptomatic AL [3]. Given the heterogeneity in rectal surgery resulting from different surgical methods and the creation of diverting stoma, we enrolled only patients who had received laparoscopic surgery without a diverting stoma to explore the risk factors for symptomatic AL.

## 2. Materials and Methods

### 2.1. Patients and Study Design

**Figure 2:**
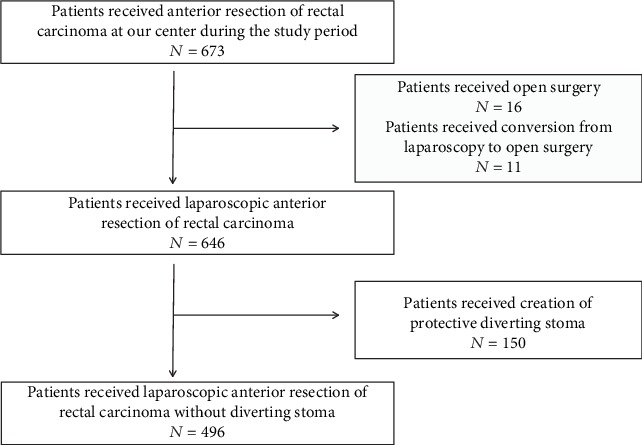
Flow chart presenting the patients' enrollment in our study.

Our study was approved by the ethics committee of our institution and was conducted following the rules of the Helsinki Declaration of the World Medical Association. All patients in our study were diagnosed with rectal carcinoma through pathological biopsy and evaluation. We searched electronic medical records from the Cancer Hospital, Chinese Academy of Medical Sciences between September 2016 and September 2017. A total of 673 consecutive patients with rectal carcinoma had received anterior resection of the rectum at our center. After excluding 16 patients who had received laparotomy and 11 patients who had undergone conversion from laparoscopy to laparotomy, laparoscopic surgery was performed on 646 cases. Among these, 150 consecutive patients received a protective diverting stoma. Eventually, 496 consecutive patients who had undergone laparoscopic anterior resection (LAR) of rectal carcinoma without a diverting stoma were enrolled in our investigation (Figure 2). We noted that the term LAR usually referred to low anterior resection of the rectum in most previous publications, which may cause confusion here. Indeed, our study included both patients who received operations above the peritoneal reflection and those who received operations below the reflection.

We retrospectively conducted a case control study in which patients who developed AL served in a case group while patients who did not were allocated to a control group. Variables regarding the demographic characteristics, living habits, comorbidities, nutritional status, preoperative chemoradiotherapy, intraoperative treatment, and tumor staging were carefully collected and analyzed between the two groups to explore the risk factors for postoperative AL.

### 2.2. Surgical Procedure

The bowel preparation was performed the day before surgery for each patient by oral administration of a sulfate-free polyethylene glycol electrolyte powder. In the operating room, the patients were placed in the lithotomy position; the pneumoperitoneum was established through intraperitoneal inflation with carbon oxide; and then the disconnection of vessels, lymph node dissection, and excision of the mesorectum were conducted through laparoscopic techniques. For patients with synchronous primary malignancy of the left hemicolon, extended resection with only one anastomosis was performed, and the resected intestine was usually longer than that of solitary rectal carcinoma. For patients who received natural orifice specimen extraction (NOSE) surgery, the specimens were extracted through the anus or vagina. For patients who received traditional LAR for the rectum, an additional small abdominal incision was created to extract the specimen. After extraction of the specimen, the pneumoperitoneum was established again, and the intestinal tract was reconstructed using a double stapling technique to form an end-to-end anastomosis. Peritoneal lavage was then routinely performed, and the air charging test was selectively carried out to evaluate the integrity of the anastomotic stoma for patients with a high risk of AL. For patients receiving intraoperative chemotherapy, the antineoplastic agents were then placed into the pelvic cavity. The available agents in our institution included lobaplatin and fluorouracil implants. Finally, one or two pelvic drainage tubes were inserted around the anastomotic stoma. For patients who were at high risk of postoperative AL, such as neoadjuvant therapy, low level of anastomosis, tissue edema, and poor blood perfusion, a transanal tube might be placed to reduce intraluminal pressure.

### 2.3. Statistical Analysis

All data were described and analyzed using the Statistical Package for the Social Sciences (SPSS version 24.0; IBM Corp., Armonk, NY). Continuous data were described as the means ± standard deviation (SD) and analyzed through a *t*-test when they were normally distributed. For continuous data that were not normally distributed, they were expressed as medians with interquartile ranges (IQRs) and further analyzed using Mann-Whitney *U* tests. Both the categorical data and ordinal data were presented as the number of cases and percentages. Categorical data were analyzed using a *χ*^2^ test or Fisher's exact tests, while the ordinal data were subsequently analyzed using Mann-Whitney *U* tests. All analyses were two-sided, and *P* < 0.05 was regarded as statistically significant. To identify the independent risk factors for symptomatic AL, multivariate logistic regression analysis was performed, and variables with a *P* value < 0.05 in the univariate analysis and factors that were reported to promote AL in previous studies were included in this model.

## 3. Results

### 3.1. Postoperative AL

A total of 496 patients were included in our investigation, with a median age of 60 years (IQR 52-66). In total, 303 (61.1%) were male and 193 (38.9%) were female. Among the 496 patients, 18 (3.6%) developed AL, and no grade A patient was enrolled in our study. Three (16.7%) AL patients were classified as grade B, whereas 15 (83.3%) were classified as grade C according to the proposal by the International Study Group of Rectal Cancer in 2010. All the 15 cases received a diverting stoma to control the abdominal infection; no cases received abdominoperitoneal resection. After that, 13 cases got their diverting stoma closed when the anastomotic stomas healed, while 2 cases did not due to their poor physical condition or anastomotic stenosis. In total, 17 (94.4%) cases developed AL within a week after surgery and 1 (5.6%) case occurred two months after the procedure (Table 1). No patients died during the perioperative period.

### 3.2. Patient-Related Variables

Patient-related variables are presented in Table 2. Patient demographics, living habits, comorbidities, neoadjuvant chemoradiotherapy, and nutritional status were compared and analyzed. We observed that AL was more likely to occur in rectal cancer patients who simultaneously suffered from a malignancy of the left hemicolon (*P* = 0.047). Other factors, including gender, age, body mass index, habits, preoperative chemoradiotherapy, and nutritional status, demonstrated no obvious association with AL.

### 3.3. Surgery-Related Variables

Surgery-related variables are presented in Table 3. All patients were treated with total mesorectal excision (TME). Most patients received traditional laparoscopic surgery in our study, and an additional incision was created for specimen extraction. One (5.6%) and 32 (6.7%) patients in the AL and control groups, respectively, underwent natural orifice specimen extraction (NOSE) surgery. NOSE surgery is an emerging surgical method in which resected specimens are extracted from the anus or vagina instead of from an auxiliary abdominal incision. The surgical approach exhibited no connection with AL occurrence. The AL group exhibited a greater proportion of patients treated by intraoperative chemotherapy compared to the control group (12 (66.7%) in the AL group versus 159 (33.3%) in the control group, *P* = 0.003). Moreover, it seemed that patients who had an anastomosis within 4 cm of the anal verge were at higher risk of AL (*P* = 0.033). Statistical analysis revealed no obvious relationship between AL and operation time, reinforcing suture, intraoperative blood loss, perioperative transfusion, preservation of left colic artery, placement of the transanal tube, and number of stapler firings.

### 3.4. Neoplasm-Related Variables

Neoplasm-related variables are presented in Table 4. Numerous previous reports have indicated that patients with low rectal carcinoma were more likely to develop AL; however, no significant relevance between AL and tumor location was observed in our study (all cases were regarded to be located above or below the peritoneal reflection based on intraoperative exploration in our study). No residual tumor was observed when pathologists examined the resected specimen in 26 patients, including 13 patients who underwent preoperative endoscopic resection and 13 patients who underwent preoperative chemotherapy or radiotherapy. AL occurred more frequently in stage T3/T4 tumors and in poorly differentiated cases, but this difference was not further confirmed in a subsequent statistical analysis.

### 3.5. Multivariate Analysis

Univariate analysis revealed that a synchronous primary malignancy of the left hemicolon (*P* = 0.047), intraoperative chemotherapy (*P* = 0.003), and the level of anastomosis (*P* = 0.033) were risk factors for AL. To adjust for confounding bias, we further enrolled these and other variables that were previously thought to increase the risks of AL in a subsequent multivariate analysis and confirmed that a synchronous primary malignancy of the left hemicolon (odds ratio (OR), 12.225; 95% confidence interval (CI), 1.764-84.702; *P* = 0.011), intraoperative chemotherapy (OR, 3.931; 95% CI, 1.334-11.583; *P* = 0.013), and level of anastomosis (OR, 3.224; 95% CI, 1.124-9.249; *P* = 0.030) were independent risk factors for AL (Table 5).

## 4. Discussion

Numerous previous studies have explored the reasons for AL after anterior resection of rectal carcinoma and performed many measures to reduce its incidence, including preventive diverting stoma, intracorporeal reinforcing sutures, preservation of the left colonic artery, placement of a transanal tube, and mobilization of the splenic flexure of the colon to decrease the tension on the anastomosis [11–16]. Moreover, several scoring systems and prediction models have been built to predict the occurrence of AL [17–19]. However, AL remains the most common and devastating issue following anterior resection of the rectum, and the incidence and risk factors for AL varied considerably in previous reports. This is probably because most of these studies included patients who had received preventive diverting stoma or patients who had received laparotomy or laparoscopic surgery. Given that preventive diverting stomas can decrease the incidence of symptomatic AL and that laparoscopic surgery has been the main operation method in most countries and areas, our study included only patients who had undergone laparoscopic surgery without a diverting stoma. Finally, a synchronous primary malignancy of the left hemicolon, intraoperative chemotherapy, and level of anastomosis were confirmed to be associated with the occurrence of AL.

Synchronous colorectal carcinoma refers to the simultaneous detection of two or more colorectal malignant lesions in a single patient at the initial diagnosis. It accounts for 1.1-8.1% of colorectal cancer, but whether it could lead to a poorer prognosis compared to solitary colorectal carcinoma is controversial [20–22]. Most previous reports have focused on exploring its risk factors and clinicopathologic features, but its impacts on surgical options and postoperative complications are less well studied. Surgical approaches depend on the distribution of synchronous cancers. For patients with synchronous carcinoma located at the rectum and left hemicolon, extended resection with only one anastomosis is the most common choice [23]. To date, only one report has demonstrated that synchronous colorectal carcinomas are a high risk factor for postoperative AL [24]. Our study included rectal cancer patients with synchronous cancer of the left hemicolon and found a significant association with AL. All of these patients underwent extended surgery, and the resected intestine was usually longer than that of solitary rectal carcinoma, which might increase the tension of the anastomoses and promote the occurrence of AL.

For patients with locally advanced rectal carcinoma, neoadjuvant therapy has been accepted as an important treatment modality to improve the clinical outcomes. However, we observed that only 18% of our patients received this therapy, which might be a result of poor economic conditions and fear of side effects. Intraoperative chemotherapy has been gradually performed in rectal cancer patients in recent years, aimed at decreasing the rate of local recurrence and distant metastasis [25]. It is generally indicated for patients with T3/T4 or N+ rectal cancer. It is conducted by placing antitumor agents into the pelvic cavity to eradicate the residual cancer cells at the end of the operation. Previous reports have confirmed that this emerging treatment modality can improve the oncologic prognosis of patients with rectal carcinoma, but its impacts on postoperative AL remain controversial [26]. Our study presented a higher proportion of patients exposed to intraoperative chemotherapy in AL patients than in non-AL patients (66.7% in the AL group versus 33.3% in the non-AL group), which was a significant difference in both the univariate and multivariate analyses This implies that intraoperative chemotherapy might be a risk factor for postoperative AL in rectal surgery. Although there have been few clinical studies on the relationship between intraoperative chemotherapy and AL, many animal studies have indicated that intraoperative chemotherapy can lead to the occurrence of AL in rats [27]. The rapid proliferation of regenerative cells is essential during the healing process of intestinal anastomoses, but implantation of antineoplastic agents can inhibit their proliferation. These agents can inhibit the activity of fibroblasts and decrease the deposition of collagen, which can reduce the mechanical strength of the anastomoses [28]. Moreover, intraoperative chemotherapy can suppress the process of vascularization, promote oxidative stress, and enhance the inflammatory response, which can contribute to anastomotic tissue necrosis [29, 30]. Surgeons need to carefully evaluate the risks of AL for patients before conducting this treatment.

Rectal cancer surgery is generally more difficult compared with colon cancer surgery given the insufficient operative view and limited working space in the pelvic cavity. The laparoscopic technique can provide an obviously better operative field than traditional laparotomy, but it also increases the difficulty of rectal transection and normally requires more stapler firings, as the cutting angle of the endolinear stapler is ineffective in this technique, and this difficulty is significantly increased when the rectum is resected at a low level [31]. Therefore, the anastomotic level has been regarded as a risk factor for AL in many previous studies, but the best cutoff value differed across reports [32]. In one systematic review that enrolled 4580 patients, the incidence of AL for patients with anastomosis levels below 5 cm from the anal verge was 8.3 times greater than the incidence for those above 5 cm [33]. In another retrospective report, the cutoff value was set as 4 cm and the AL rate was 6 times higher when the anastomosis was located within 4 cm of the anal verge [34]. In our study, we observed that an anastomosis level ≤ 4 cm was significantly associated with the occurrence of AL (5.9% versus 2.3%). This can be attributed to the increased difficulties in performing the technique as the anastomotic distance from the anal verge decreases. In addition, a poorer blood supply caused by a lower anastomotic level might be another reason for the high risks of AL. We also explored the relationship between AL and tumor location, but no statistically significant differences were observed, which was in line with some previous reports [35]. Given the difference about the length of the distal margin from the low border of the rectal tumor between tumors located above and tumors located below the peritoneal reflection, we believe the level of anastomosis may be a better index to predict AL than tumor location.

Given the serious consequence of AL, most surgeons tend to choose the creation of a preventive diverting stoma for patients with high risks of AL. Our study excluded 150 (22.3%) patients who received a diverting stoma; it is unclear how many of them truly benefited from this procedure. Many of them may not develop AL even if they do not have a prophylactic stoma, and it brings patients risks of stoma-related complications and a second operation to close it. Surgeons need to think carefully about this for patients. Neoadjuvant therapy, low level of anastomosis, tissue edema, and poor blood perfusion are mostly believed to lead to AL. In our center, patients with two or more of these risk factors need to receive a diverting stoma. Moreover, for patients with risky distrustful anastomoses, such as a positive result of air charging test and incomplete incisal margin from staplers, they will undergo protective stomas even if no risk factors exist.

Our study has the following limits. First, the retrospective nature of our study makes the bias from patient selection and data collection difficult to avoid. Second, the incidence of AL in our report is much lower than that in most previous reports, and the limited number of patients in the AL group might hinder the findings of more risk factors for symptomatic AL in our study.

## 5. Conclusions

In conclusion, our present research determined that synchronous primary malignancy of the left hemicolon, intraoperative chemotherapy, and low anastomotic levels were independent risk factors for symptomatic AL after LAR for rectal carcinoma.

## Figures and Tables

**Table 1:** AL patients.

	AL patients (*n* = 18)
AL grade, *n* (%)	
A	0 (0%)
B	3 (16.7%)
C	15 (83.3%)
Occurrence time of AL, *n* (%)	
Early AL	17 (94.4%)
Delayed AL	1 (5.6%)

AL: anastomotic leakage.

**Table 2:** Patient-related variables.

Variables	AL (+) (*n* = 18)	AL (−) (*n* = 478)	*P*
Age (yr)			
Median (IQR)	56 (50.75, 60.5)	60 (52, 66)	0.297
Sex, *n* (%)			0.139
Male	14 (77.8%)	289 (60.5%)	
Female	4 (22.2%)	189 (39.5%)	
BMI (kg/m^2^, mean ± SD)	24.0 ± 3.3	24.1 ± 3.5	0.853
Smoking, *n* (%)	6 (33.3%)	135 (28.2%)	0.638
Alcohol, *n* (%)	6 (33.3%)	104 (21.8%)	0.383
Hypertension, *n* (%)	4 (22.2%)	128 (26.8%)	0.875
Ischemic heart disease, *n* (%)	0 (0%)	15 (3.1%)	1.000
Diabetes, *n* (%)	2 (11.1%)	54 (11.3%)	1.000
Hepatitis, *n* (%)	1 (5.6%)	27 (5.6%)	1.000
History of malignancy, *n* (%)	0 (0%)	17 (3.6%)	1.000
Synchronous primary malignancy of left hemicolon, *n* (%)	2 (11.1%)	8 (1.7%)	0.047
Incomplete intestinal obstruction, *n* (%)	2 (11.1%)	41 (8.6%)	1.000
Preoperative chemotherapy, *n* (%)	3 (16.7%)	58 (12.1%)	0.834
Preoperative radiotherapy, *n* (%)	1 (5.6%)	27 (5.6%)	1.000
Preoperative hemoglobin (g/L, mean ± SD)	137.7 ± 17.4	137.3 ± 17.3	0.830
Preoperative albumin (g/L, mean ± SD)	43.2 ± 3.7	44.2 ± 3.6	0.247
ASA grade, *n* (%)			0.620
1	0 (0%)	18 (3.8%)	
2	17 (94.4%)	433 (90.6%)	
3	1 (5.6%)	27 (5.6%)	

AL: anastomotic leakage; IQR: interquartile range; SD: standard deviation; BMI: body mass index; ASA: American Society of Anesthesiologists.

**Table 3:** Surgery-related variables.

Variables	AL (+) (*n* = 18)	AL (−) (*n* = 478)	*P*
Natural orifice specimen extraction surgery, *n* (%)	1 (5.6%)	32 (6.7%)	1.000
Operation time (min, mean ± SD)	171.6 ± 45.4	162.5 ± 62.4	0.206
Consolidation suture, *n* (%)	1 (5.6%)	71 (14.9%)	0.448
Intraoperative chemotherapy, *n* (%)	12 (66.7%)	159 (33.3%)	0.003
Estimated blood loss (mL, mean ± SD)	56.7 ± 34.0	65.1 ± 88.0	0.506
Transfusion, *n* (%)	1 (5.6%)	22 (4.6%)	0.641
Left colic artery preservation, *n* (%)	1 (5.6%)	39 (8.2%)	1.000
Transanal tube, *n* (%)	7 (38.9%)	270 (56.5%)	0.140
Anastomotic level from anal verge (cm)			0.033
≤4	11 (61.1%)	174 (36.4%)	
>4	7 (39.9%)	304 (63.6%)	
Number of stapler firing, *n* (%)			0.819
1 and 2	13 (72.2%)	370 (7740%)	
Greater than 2	5 (27.8%)	108 (22.6%)	

AL: anastomotic leakage; SD: standard deviation.

**Table 4:** Tumor-related variables.

Variables	AL (+) (*n* = 18)	AL (−) (*n* = 478)	*P*
Tumor location, *n* (%)			0.602
Above peritoneal reflection	13 (72.2%)	383 (80.1%)	
Below peritoneal reflection	5 (27.8%)	95 (19.9%)	
Pathological T stage, *n* (%)			0.246
Tis, T1, T2, and no tumor residual after preoperative therapy	3 (16.7%)	140 (29.3%)	
T3 and T4	15 (83.3%)	338 (70.7%)	
Pathological N stage, *n* (%)			0.854
N0 and no tumor residual after preoperative therapy	10 (55.6%)	255 (53.3%)	
N1 and N2	8 (44.4%)	223 (46.7%)	
Pathological M stage, *n* (%)			0.553
M0	18 (100%)	448 (93.7%)	
M1	0 (0%)	30 (6.3%)	
TNM stage, *n* (%)			0.746
0-II and no tumor residual after preoperative therapy	10 (55.6%)	247 (51.7%)	
III-IV	8 (44.4%)	231 (48.3%)	
Degree of differentiation, *n* (%)			0.337
Low, low-middle grade	7 (38.9%)	136 (28.5%)	
Middle, high-middle, high grade, and no tumor residual after therapy	11 (61.1%)	342 (71.5%)	

AL: anastomotic leakage.

**Table 5:** Multivariate logistic regression analysis.

Variables	*P*	OR	95% CI
Gender	0.095	2.742	0.841-8.943
Age	0.723	1.009	0.962-1.057
BMI	0.644	1.033	0.899-1.188
Diabetes	0.721	1.332	0.276-6.421
Synchronous primary malignancy of left hemicolon	0.011	12.225	1.764-84.702
Preoperative radiotherapy	0.753	1.415	0.163-12.282
Intraoperative chemotherapy	0.013	3.931	1.334-11.583
Perioperative transfusion	0.400	2.561	0.287-22.887
Level of anastomosis	0.030	3.224	1.124-9.249
Transanal tube	0.171	0.486	0.173-1.364
Number of stapler firing	0.852	0.895	0.279-2.868

BMI: body mass index; OR: odds ratio; CI: confidence interval.

## Data Availability

The data used to support the findings of this study are available from the corresponding author upon request.
